# A phenomenologically grounded specification of varieties of adolescent depression

**DOI:** 10.3389/fpsyg.2024.1322328

**Published:** 2024-02-23

**Authors:** H. Andrés Sánchez Guerrero, Ida Wessing

**Affiliations:** Department of Child and Adolescent Psychiatry, Psychosomatics, and Psychotherapy, University Hospital Münster, Münster, Germany

**Keywords:** adolescence, depression, experience, personality structure, phenomenological psychopathology

## Abstract

Researchers are increasingly acknowledging that psychopathological conditions usually grouped together under the generic label “depression” are highly diverse. However, no differential therapeutic approach currently exists that is sensitive to the varieties of depression afflicting young people. In fact, the discussion is missing something much more fundamental: a specification of the types of adolescent depression. Recent research that has aimed to classify different kinds of depression has mainly studied adult populations and predominantly used technically complicated measurements of biological markers. The neglect of the potential particularities of dysphoric disorders affecting youths is unfortunate, and the exclusive focus on biological parameters unnecessarily restrictive. Moreover, this one-sidedness obfuscates more directly available sources of clinically relevant data that could orient conceptualization efforts in child and adolescent psychiatry. Particularly, clues for discriminating different types of adolescent depression may be obtained by analyzing personally articulated accounts of how affected young people experience changes in their relation to the world and to themselves. Thus, here we present and discuss the findings of a study that explored the possibility of specifying types of adolescent depression in a phenomenological way. The study investigated the association between these types and the vicissitudes of personality development. In accounts given by youths diagnosed with depression during semi-structured interviews, we identified themes and examined their phenomenological centrality. Specifically, our qualitative analyses aimed to determine the relative importance of certain themes with respect to the overall intelligibility of the described changes to the relational space. Based on the findings of these analyses, we differentiate three specifiers of adolescent depression and suggest an association between particular types of experiences and the trajectory of affected adolescents’ personality development. To our knowledge, this is the first phenomenologically grounded specification of types of adolescent depression with potential therapeutic significance. Thus, based on this contribution, we propose that modes of scientific exploration that are close to phenomenological philosophy—which have been ignored in the context of developmental psychopathology—could offer a foundation to theories developed in the field of child and adolescent psychiatry.

## 1 Introduction

The most recent revision of both international manuals for the classification of mental disorders [DSM-5 (American Psychiatric Association, 2013) and ICD-11 (World Health Organization, 2019/2021)] retained the expression *depressive disorders* to designate a domain of episodically occurring—and often recurrent—symptoms associated with a dysphoric mood. This use of a plural term may be an attempt to acknowledge the diversity of conditions grouped together under the generic label *depression*. However, since the “operational revolution” of psychiatric nosology—made official with the publication of the DSM-III (American Psychiatric Association, 1980)—psychiatric research has primarily focused on so-called major depressive disorder, as if major depression constitutes a prototype that allows for understanding how to conceptualize, assess, treat, and prevent *all* clinically relevant dysthymic conditions.^1^

In recent years, many authors have reminded us, though, that the category *major depression* emerged in the course of a move that clustered together two forms of longstanding dysphoric suffering which, up to that point, had been systematically separated; these two forms were called melancholic and non-melancholic depression (cf. Shorter, 2007; Kendler, 2020; Kim and Park, 2020). Here, it remains controversial whether this “classical” dichotomy comprised exactly two types of dysphoria, since non-melancholic depression had been historically contoured in a purely negative way—as a mere contrast to melancholia—suggesting that it might encompass multiple types of dysthymic disorders. Thus, combining these two types of depression ultimately had the outcome, as noted by Shorter, of creating “a single depression category into which almost any dysphoric patient could be squeezed” (Shorter, 2007, p. 10).

To complicate the matter, the heterogeneity of the conditions subsumed under the term *depressive disorder* may be immanent to the polythetic approach underlying both classification systems, where the diagnostic criteria encompass diverse constellations of symptoms that are considered to be nosologically equivalent. For instance, using the DSM 5, Kim and Park (2020) calculated that the criteria for major depressive disorder can be fulfilled via 227 combinations of symptoms. Hence, in “masking” heterogeneity through collapsing diverse dysthymic states into a single category, modern psychiatry may have created conditions that can explain why clinical practice sees substantial inter-individual differences in patients’ responses to “treatments for depression” (cf. Fried and Nesse, 2015).

Clinically relevant longstanding dysphoric predicaments afflicting children and adolescents are probably even more multifarious. For decades, researchers and clinicians have embraced the idea that depressive symptomatology shows a “heterotypic continuity” (Kagan and Moss, 1962) across ontogenetic development. In this context, the notion has become prevalent that, in the pediatric population, mood lability or irritability and a series of behavioral manifestations—as distinct from a reported low mood—could be regarded as principal symptoms of depression (cf. Mueller and Orvaschel, 1997; Crowe et al., 2006; Cook et al., 2009). While initial evidence has suggested that this notion is contestable (cf. Stringaris et al., 2013), the ongoing discussion mirrors the clinical impression that the varied conditions classified as depression at a young age are extremely miscellaneous in character. Thus, Midgley and coauthors emphasize that “the nature of adolescent depression is still not sufficiently understood, with the psychiatric concept of ‘depressive disorders’ covering a heterogeneous range of difficulties” (Midgley et al., 2015, p. 270).

Even if the cardinal symptoms of depression are identical for adolescents and adults, one could argue that current psychodiagnostic manuals ignore key aspects of afflicted adolescents’ experiences (cf. Lachal et al., 2012)—experiences that are often related to problems that cause significant impairment and endanger these adolescents’ further development. In this context, studies based on in-depth interviews of depressed young people have tried to identify the main motifs associated with “experiences of adolescent depression.” In a British sample of 77 adolescents, Midgley et al. (2015) distilled the following five themes: misery, despair, and tears; anger and violence toward self and others; a bleak view of everything; isolation and cutting off from the world; and a negative impact on education. In a U.S. American sample of five adolescents, Farmer (2002) identified three central motifs, namely anger, fatigue, and interpersonal difficulties. Finally, in a German sample of six adolescents, Weitkamp et al. (2016) extracted the following four key themes: suffering that is experienced as overwhelming, an experience of loneliness and isolation, struggling to understand one’s suffering, and therapy as a last resort.

The diverse sets of motifs identified by these researchers may share commonalities. In a meta-synthetic study including six qualitative investigations, Dundon (2006) identified the following five themes across diverse studies: struggle to make sense of the situation, social withdrawal, self-harm as an attempt to deal with the situation, suicidal ideation, and engagement in risky behavior. However, taken together, the results of these analyses might reinforce the impression that adolescent depression is, also at the level of subjective experiences, highly variable. Moreover, these distilled themes could also be expected to arise in the personal accounts of adolescents suffering other psychiatric conditions.

Given this clinical and experiential multifariousness, critics of the current conceptualization have claimed that depression should be understood as a domain of symptoms rather than a singular psychopathological process (cf. Parker, 2014). Accordingly, recent scientific work has aimed to develop novel classifications for types of depression. This work has focused on adult populations and on technically complicated measurements of biological markers, such as compound profiles of hormones and other molecules (e.g., leptin, brain-derived neurotrophic factor, tryptophan), functional neuroimaging patterns, estimations of genetic heterogeneity, and correlations of neuroimaging and genetic data (cf. Beijers et al., 2019; Lynch et al., 2020; Buch and Liston, 2021; Nguyen et al., 2022).

In our view, neglecting the potential particularities of dysthymic predicaments that are affecting young people is unfortunate, and exclusively focusing on biological parameters is unnecessarily restrictive. This one-sidedness, we believe, obfuscates more directly available sources of clinically relevant data that could orient conceptualization efforts in child and adolescent psychiatry. Particularly, analyzing personal accounts may be highly lucrative, as the experiences described by adolescents diagnosed with depression are not simply heterogeneous. Notably, certain descriptive motifs seem also to be inter-individually recurrent. Drawing on the intuition that these motifs may, furthermore, be *differentially* recurrent across individuals, such personal accounts could be expected to offer clues to discriminate the varieties of adolescent depression.

This contribution presents and discusses the findings of an exploratory study that examined the plausibility of this intuition and investigated a possible association between varieties of adolescent depression and the vicissitudes of personality development. This inquiry drew on qualitative analyses of in-depth interview transcripts focusing on the experiences of adolescents diagnosed with depression. Our exploration aligned with the mode of investigation called phenomenology—a form of philosophical inquiry that illuminates reality by disclosing the intentional constitution of the *life-world* [*Lebenswelt*] (Husserl, 1936/1970). Departing from a descriptive characterization of the ways in which the world appears in everyday life, a phenomenological exploration reveals a series of meaning-bestowing acts that may be regarded as structural moments of experience. Thus, our interpretative analyses aimed to determine the importance of certain descriptive motifs with respect to the overall intelligibility of a series of changes in participants’ relation to the world and to themselves. In closing this introductory discussion, we specify the sense in which we understand our inquiry as an exercise in phenomenological psychopathology. [A detailed discussion of theoretical premises, design, instruments, and interpretative procedure of this study is offered elsewhere (Sánchez Guerrero, 2023).]

We use the term *phenomenological psychopathology* to refer to investigations that, drawing on descriptions provided by individuals diagnosed with a psychiatric disorder, expose “constitutive moments” that determine boundaries of comprehensibility of certain experiences associated with a psychopathological condition. These studies discriminate experiential world domains that in the relevant condition typically undergo fundamental changes, presumably due to morbid processes. Concerning specifically “experiences of depression,” for instance, Ratcliffe (2015) conceived of these transformations in terms of an altered sense of the space of possibilities.

To implement an empirical approach oriented by this understanding of a phenomenological-psychopathological exploration, we adapted the principles of a widely used qualitative research method called interpretative phenomenological analysis. Yet, we do not take the phenomenological character of our exploration to be rooted in our use of this empirical method; rather this character arises from our goal to illuminate life-world transformations associated with adolescent depression by analyzing structural moments of the described changes. Specifically, our examination draws on the notion that some of the constituents of the experiential field—the multilayered edifice of interrelated meaning-components that make up the world as experienced—play a structuring role (while others are, correspondingly, structured by the former).

We conceived of this structuring-structured relationship in terms of the capacity that certain fragments of an analyzed personal account have to serve as *conditions of intelligibility* to other fragments of the same account. Our goal was to expose relatively central aspects of a meaning-complex that is expressed in singular but (presumably) interrelated descriptions that constitute a particular personal account of adolescent depression.

Based on the results of our analyses, this paper proposes a phenomenologically grounded specification of varieties of adolescent depression, which, as we claim, has potential therapeutic significance. This claim of conceivable clinical relevance is founded on a relationship between the specified modes of adolescent depression and our participants’ personality development trajectory. Here, we propose this association on the basis of a purely descriptive (i.e., non-statistical) pairing of two sets of results. This proposal aligns with findings concerning a “borderline form” of depressive experience reported in the literature (for a systematic review, see Köhling et al., 2015).

To our knowledge, no comparable study has yet been published that explores the role that different experiential meaning-structures play in constituting the “world of depression” (Ratcliffe, 2015) as described by affected youths. Correspondingly, no attempt has yet been made to specify varieties of adolescent depression in a phenomenological manner. Thus, our findings, which partially challenge the “received view,” may help refine the ways in which we clinically assist adolescents suffering from impairing dysphoric conditions. Particularly, our results suggest a possible direction for assessments that meet the desideratum of a differential therapeutic indication sensitive to the notion that adolescent depression “is not just adolescent depression.” This may, in turn, dissipate the prevailing assumption of treatment “equipotency,” which, as Parker put it, “promotes therapeutic eclecticism” (Parker, 2014, p. 404). Such a move would align with the spirit of personalized medicine.

The structure of our report is as follows. After describing the characteristics of the sample (Subsection 2.1) and the procedures by means of which we generated the data (Subsection 2.2), we describe our systematic of interpretation to highlight this approach’s key attributes (Subsection 2.3). In Section 3, we initially present our results in an abstract way that allows us to propose a classification of varieties of adolescent depression in terms of three phenomenological specifiers (Subsection 3.1). Subsection 3.2 explicates our classificatory decisions and illustrates relevant points through concrete verbatim material and intermediate results of the analyses. In Subsection 3.3, we put in relation different sets of results. The discussion in Section 4 touches on the comparability of our findings with those of related studies (Subsection 4.1), the particular transformation of structures of experience suggested by our qualitative analyses (Subsection 4.2), the link between our specifiers and certain types of depression proposed elsewhere (Subsection 4.3), a conceivable relationship between the worked-out phenomenological specifiers and the trajectory of personality development (Subsection 4.4), possible future directions suggested by our results (Subsection 4.5), and the most important limitations of this study (Subsection 4.6).

## 2 Materials and methods

### 2.1 Sample

Participants were young people aged 14–17 years who, following a clinical assessment at the Department of Child and Adolescent Psychiatry of the University Hospital Münster, fulfilled ICD-10 criteria for diagnosing a depressive episode. As such, they reported a minimum of four of ten possible symptoms stipulated in ICD-10 that had persisted for 2 weeks or longer, where reduction in energy counted as an entry-level symptom. A child and adolescent psychiatrist or a trained clinical psychologist conducted this assessment. We did not employ any structured clinical interview to secure the diagnosis. However, our participants were required to estimate the severity of their symptoms by means of the Beck Depression Inventory (BDI-II; A. T Beck, R.A. Steer, and G.K. Brown). In this way, we obtained a rough indicator that, at the point of recruitment, the symptoms had at least the clinical significance of a mild depressive episode (i.e., a score of 14 points or more).

Sufficient for eligibility was an adolescent having gone through a depressive episode in the context of one of the mixed conditions classified in ICD-10 (e.g., F41.2 or F92.0). Most forms of psychiatric comorbidity—previous or current—did not constitute a reason to exclude potential participants. Exclusion criteria were only met in cases of acute suicidality, impaired insight (particularly in psychotic states), or when proficiency in the German language appeared insufficient to enable participation in two interviews described below. The inclusive character of our recruitment reflected our interest in getting a representative sample of the heterogeneous group of adolescents with “symptoms of depression” habitually referred to a specialized mental health service.

A group of 10 adolescents constituted the sample of the study. Seven participants were female and three were male, which maps the reported proportion of female to male adolescents stating symptoms of depression (cf. Breslau et al., 2017). Their mean age was 15,5 years. Some participants’ clinical characteristics are summarized in Table 1.^2^

**Table 1:** Participants’ characteristics.

Participant	Psychiatric diagnosis justifying inclusion (ICD-10)	Additional psychiatric diagnoses (ICD-10)	Antidepressant treatment	Outpatient psychotherapeutic treatment	Hospitalization (incl. day-care hospital)
#1	Other depressive episode (F32.8)	Social phobia (F40.1)	Yes (current)	Yes (current)	Yes (previous)
#2	Depressive conduct disorder (F92.0)	Other childhood emotional disorder (F93.8)*	No	No	Yes (previous)
#3	Moderate depressive episode (F32.1)	Other childhood emotional disorder (F93.8)	No	No	No
#4	Recurrent depressive disorder, current episode moderate (F33.1)	Panic disorder (F41.0) Undifferentiated somatoform disorder (F45.1)	No	No	No
#5	Moderate depressive episode (F32.1)	Other childhood emotional disorder (F93.8)	Yes (previous)	Yes (current)	No
#6	Depressive conduct disorder (F92.0)	Panic disorder (F41.0) Post-traumatic stress disorder (F43.1)	No	Yes (previous)	Yes (previous)
#7	Moderate depressive episode (F32.1)	None	No	No	No
#8	Moderate depressive episode (F32.1)	Panic disorder (F41.0)	No	Yes (previous)	No
#9	Moderate depressive episode (F32.1)	Other childhood emotional disorder (F93.8)	No	Yes (previous)	Yes (current)
#10	Mixed anxiety and depressive disorder (F41.2)	None	No	Unclear	Yes (current)

*The vague category F93.8 was employed to codify affect regulation problems that seemed to not be restricted to the described depressive episodes.

### 2.2 Data generation

In the course of an open-ended thematically centered interview (Depression Experiences Interview [DEI]), these participants articulated in words what is special about specific aspects of their world- and self-related experiences when they are going through a depressive episode. The Depression Experiences Interview Schedule (DEIS)—developed for this study—guided the semi-structured conversations with the participants. The DEIS empirically implements an approach to certain structural transformations of the experiential field that have been described in extant phenomenological-psychopathological literature on depression (cf. Minkowski, 1933/2019; Binswanger, 1960; Tellenbach, 1961/1980; Fuchs, 2013; Ratcliffe, 2015; for a discussion of the structure of the DEIS, see Sánchez Guerrero, 2023).

Through a second semi-structured conversation based on the Axis Structure interview schedule of the second version of the Operationalized Psychodynamic Diagnosis in Childhood and Adolescence (OPD-CA-2) instrument (OPD-CA-2 Task Force, 2017), we assessed participants’ personality structures. The OPD-CA-2 conceives of personality development along a continuum of levels of structural integration. This continuum extends from good integration, to limited integration, to low integration, to disintegration. These levels are scored from 1 to 4. Structural integration is assessed in relation to four dimensions: control, identity, interpersonality, and attachment. Each dimension is operationalized in structural abilities, which the rater is expected to assess in relation to anchor-point descriptions specified for each level of integration. One of the coauthors (I.W.), who was blind to all information concerning the participants (except for their age), rated the audio recordings obtained during the OPD-CA-2 interviews. Most notably, the OPD-CA-2 rater (I.W.) was blind to the personal accounts of depression obtained during the Depression Experiences Interview (DEI). She did not know the participants, and she was not involved in their diagnostic or therapeutic processes. All rated interviews were conducted by the other coauthor (H.A.S.G.), who had, until analyzing the DEI transcripts, remained blind to the OPD-CA-2 rating results.

Additionally, we estimated the participants’ identity development by means of the self-assessment questionnaire Assessment of Identity Development in Adolescence (AIDA; K. Goth and K. Schmeck). The AIDA conceives of the vicissitudes of identity development along a single dimension starting from a healthy identity, to normative identity crises, to pathological identity diffusion. It differentiates an affective dimension of identity pathology concerning the degree of discontinuity of a person’s experience of self-sameness from a cognitive dimension pertaining to the degree of incoherence of her image of herself and others. Higher scores are associated with a (higher risk of developing a) personality disorder.

Participants and their caregivers provided written informed consent before we conducted the interviews and the AIDA assessment. The Ethics Committee of the Medical Council Westfalen-Lippe had approved this design.

### 2.3 Data analysis (transcript interpretation and non-statistical pairing)

We analyzed the DEI transcripts in an idiographic manner. In interpreting the accounts, we drew on the notion that the experiential world of adolescent depression can be understood as a multilayered field of interrelated meaning structures. Our analysis aimed to reduce the complexity of this web of connotative structures. We achieved this by identifying and hierarchically arranging thematic motifs.

As mentioned above, we elaborated on principles of the qualitative research method interpretative phenomenological analysis (IPA), as described by Smith and Osborn (2015). As argued elsewhere (cf. Sánchez Guerrero, 2023), this method permits us to (1) expose a complex of meaning horizons that appear fundamental to the comprehensibility of the reported lived experiences and (2) determine the structural character of the meaning of certain fragments of an analyzed personal account. We proceeded as follows.

Convergent themes were grouped together and repositioned within the relevant group with respect to what we call their *phenomenological centrality*, which refers to a motif’s importance with respect to the overall comprehensibility of the described experiential field changes. The outcome was a ranking of thematic bundles (cf. Supplementary material).

Here, we determined a motif’s position in the hierarchy by assessing how well the motif was able to illuminate other themes of the same bundle by offering a more general/abstract picture of the relevant matter. Specifically, within an emerging thematic bundle, we gave a higher ranking to a motif that offered a broader frame of comprehensibility to other themes within that bundle. As such, we understood those lower-ranked themes to be concretizations of a more wide-ranging motif. Therefore, the theme ranked the highest within an emerging bundle was considered the motif with the highest capacity to confer general intelligibility to the relevant bundle.

A hierarchy of the distinct emerging bundles was determined following the same logic. Thus, we regarded the heading of the very first bundle as the theme that was most apt to confer overall intelligibility to the relevant account as a whole. Put another way, the heading of the first bundle captures the most comprehensive condition of intelligibility of the various topics that constitute the analyzed account.

Notably, this procedure, which allows for determining the level of phenomenological centrality of identified themes, does *not* weight the importance of a theme according to how frequently it appears within a transcript.

From these idiographic analyses of the DEI transcripts, accounts that touched on similar issues were grouped together. The goal of this categorization was not to determine mutually exclusive types; thus, even if an account was classified under one of the emerging groups, the account may still contain characteristics related to a different group (see the discussion in Subsection 4.1). However, to be able to describe relatively contrasting abstract specifiers, each account was classified into exclusively one group.

An intragroup comparative analysis allowed us to identify thematic bundles that appeared *typical* of each relevant group, while an intergroup comparative procedure allowed us to distill themes that were *specific* to a particular group. On this basis, we worked out the three ideal-typical phenomenological specifiers described in the next section. [An illustration of the different steps of the procedure can be found in Sánchez Guerrero (2023).]

In a last step, we explored a possible association between the specifiers we worked out and the personality development trajectory as assessed by the above-mentioned instruments. We did this through a tabular juxtaposition of those results that we wanted to put in relationship.

## 3 Results

### 3.1 Emerging phenomenological specifiers

This subsection succinctly depicts what our results suggest is distinctive of three discriminated varieties of adolescent depression. This abstract level of description should facilitate comparison by providing ideal-typical characterizations of these varieties (Boxes 1–3). To extend and make these abstract characterizations more concrete, Subsection 3.2 includes verbatim text and results of intermediate analytic steps.^3^

The majority of interviewed young people (6/10) described a predicament we would like to call the *Separated (Yearning) Mode* of adolescent depression (Box 1).

BOX 1 **The separated (yearning) mode.**

This form of adolescent depression is characterized by a painful longing for a sense of familiarity with a world from which the affected adolescents feel to have been disconnected.

The world of adolescents affected in this way remains in a sense intact. However, these youths experience it as a space that has lost its habitual character and from which they have somehow been excluded—as a realm they can no longer participate in. This leads to a distressing sense that the experiential field has become self-discrepant. This paradoxical experience of “separated-being-in” correlates with the stressful feeling of being captive in loops of affects and thoughts.

A sense of alienation is a cardinal part of the described experiences, particularly in relation to arising life-negating and self-destructive impulses. Affected adolescents feel disoriented and pulled in different directions by an urgent drive to get out of this predicament, which painfully coexists with a sense of lacking the power, interest, and motivation necessary to change their situation. Thus, another experienced paradox characteristic of this dysphoric condition concerns the feeling of having to go through “a life that is devoid of vitality.”

Affected adolescents often feel misunderstood, especially by their peers, and want to avoid contact. Even though they describe an unbearable loneliness, they feel like they have to retreat further. Usually, though, relationships with significant adults are experienced as exhibiting positive aspects.

A smaller subgroup of participants (2/10) portrayed a condition that might be characterized as the *Upset (Refusing) Mode* of adolescent depression (Box 2).

BOX 2 **The upset (refusing) mode.**

Adolescents acquainted with this kind of dysphoric experience report phases in which a rejecting attitude, which they feel unable to modify, makes it difficult to accept anything that the world is disposed to offer as positive or comforting.

This declining attitude goes hand in hand with a feeling of demotivation, powerlessness, and reduced capability to enjoy activities as well as with a sense of not being able to find meaning in everyday routines that are, correspondingly, experienced as boring. Sufferers experience themselves as irritable and emotionally labile.

Even though they fear being alone, affected adolescents feel that, depending on behaviors of significant others which they register as hurtful, they have to withdraw from the social world and close themselves off. It is as if they have to refuse doing anything that could be understood as a form of cooperation with an offensive world. This predicament situationally leads to a desperate wish to die or even to an urgent impulse to commit suicide.

To these adolescents, caregivers often seem to be annoyed by them. Many interactions are encumbered by a feeling of self-abomination, which generates a kind of distrust in relationships.

Finally, a third subgroup of participants (2/10) portrayed a form of adolescent dysphoria that we labeled the *Paralyzed (Non-Resonating) Mode* of depression (Box 3).

BOX 3 **The paralyzed (non-resonating) mode.**

This form of world-and self-relatedness is articulated in terms of a peculiar form of apprehension comprising long-term periods during which the affected adolescents feel apathetic, lethargic, unable to resonate with the world, and practically paralyzed.

Affected adolescents describe a sense of being rather insensitive and indifferent, as opposed to being in some particular (negative) mood. A feeling of being overworked, indescribably exhausted, and virtually unable to move constitutes a central feature of this predicament.

The sufferers have the impression that this prolonged state amounts to a sort of pseudo-immobility that can, however, completely incapacitate. This impression is the foundation of the experiential self-discrepancy that is typical of this form of longstanding adolescent dysphoria. This confusing sense of no longer being an agent, despite being, in principle, able to act, is associated in a circular way with a feeling that life is immutable and worthless. Thus, the focus of the affected adolescents’ rumination is the conviction that the “opportunities” they are confronted with do not amount to real possibilities, but merely to non-appealing possible realities. This leads to a sense of tragic tolerance of an existence that seems to have no point.

In these periods, other people, especially authority figures, are often perceived as hostile or unfair, since they seem to hardly be able to understand the radical transformation of life that is associated with this sense of pseudo-paralysis.

Note that all three specifiers describe a sort of experienced contradiction that is integral to the very way in which affected young people find themselves in the world. Therefore, these three characterizations are thought to capture the specific *existential incongruity* that fits with each of the distilled modes of adolescent depression.

### 3.2 Some hallmarks of the identified varieties (explicative characterization)

Seeking to point to a set of markers of the Separated (Yearning) Mode, one could mention two features. The first pertains to a sense of self-estrangement that is associated with a feeling of having lost familiarity with the world. The second concerns a distinct contextual dependence of the reported experiences of social disconnection that leaves caregiving figures untouched.

Youths whose accounts contained the first feature articulated a feeling that, during the relevant episodes, some of their thoughts, experiences, and/or acts did not correspond to the way they “actually” tended to think, perceive, and/or behave. It is as if they had been slightly shifted away from the space of reason, perception, and action they were familiar with. This was experienced as a distortion and a limitation of their proper way of being. Participant #10 described it as follows:

Yes, well, sometimes when I am in this state, I have different views or I am so restricted in thinking. I can barely …, well, somehow, it’s as if you have these glasses on through which you definitely don’t see certain things anymore, but it’s so completely …, it turns away from the logical picture of the world and …, entertains such a weird, completely twisted perception or thinking.

This alienation concerning the way they had come to perceive, think, and act could be regarded as “the other side” of the mentioned sense these adolescents reported, to the effect that the world had lost its habitual character. In conjunction with a felt lack of power and motivation, these experiences of (self-)estrangement led to a recurrent feeling that life had puzzlingly become bereft of vigor—a feeling that may also be characterized in terms of a world that had become unlikely to arouse certain impulses and responses.

Concerning the second feature, it is important to note that most of our participants expressed a painful feeling of solitude and separation as well as a sense of being profoundly misunderstood. However, only those participants whose accounts were classified under the Separated (Yearning) Mode specifier described these feelings particularly in relation to their peers. Participant #7, for instance, said, “I cannot really associate with them …, it seems very strange to me to talk to people my age …. They make me feel the least understood, […] the furthest away.” In a similar vein, participant #8 answered as follows the question as to how she perceives her peers during the episodes at issue: “I cannot describe it at all, but they definitely seem to me like … that sounds weird, but like bad people, for instance, so they seem to me like they are my enemies or … the opposite of me, actually.” Interestingly, both participants described a largely unmodified relatedness to adults.

Two participants’ accounts (participant #2 and participant #6) were grouped into the Upset (Refusing) Mode of adolescent depression, for which three hallmarks were identified: (1) a gravitation of central themes around a vaguely articulated sense of not being wanted, which is associated with a rejecting attitude; (2) a background sense of being upset, which is either situationally expressed in aggressive behavior or, in other phases, transformed into profound sorrow or masked by a feeling of numbness; and (3) an extreme dependence of these participants’ mood on changes in the perceived social situation associated with a feeling of having been rejected or invalidated.

For the first hallmark (a vaguely articulated sense of not being wanted), the following description of participant #6 succinctly reflects this feeling: “Well, you just do not feel right here anymore. So, I do not know what it is …, you just do not feel cared for anymore.” This feeling of not being an object of care/love—which can take the form of a feeling of having been neglected, disrespected, or offended in some usually unclear way—is associated with the declining attitude that we captured under the notion of a refusing mode of depression. This attitude, which involves a reactive closing off from other people’s attempts to come close, was described as involving an aspect of self-destructivity and/or disruptiveness.

Concerning the first hallmark, in relation to participant #2’s account, we worked out a first thematic bundle related to the feeling that the social world had thrown her into the realm of depression by causing her to express persistent sadness and negativity; this bundle was called “Focusing on the sad and negative that has been imposed on you.” We captured the refusing and potentially disruptive attitude in the third bundle’s title: “Injury, defiance, and (identification with) destruction.” This bundle was partially derived from descriptions that conveyed how, when she felt neglected or maltreated, participant #2 usually exhibited a relatively generalized oppositional attitude: “Yes, then I do not want to go to school, I do not cooperate, I do not allow others to tell me how to do things.”

In a similar vein, in relation to participant #6, we arrived at a first bundle related to the topic of a world that was increasingly less apt to lift her up, which we associated with the first hallmark (a sense of not being an object of care/love). Trying to capture the agonizing feeling that she might be a burden to others and that important people would not need her—a feeling that led her to reject contact, become skeptical of relationships, and denigrate herself—we entitled the second bundle “A self-loathing that becomes paranoid: self-depreciation and distrust in relations.” Here is participant #6’s description of a deeply skeptical attitude which she developed against her closest friends, specifically:

Well, with my normal friends it’s just that they can come and go, so, I don’t care, they can go and stay away. But with my closest friends […], I don’t know, I have the feeling that they suddenly become so devious that I couldn’t trust them anymore, that I just … I don’t know, it’s just like that … if they were there at the moment when I’m feeling so bad that I really got to the point where my friends then change in my eyes, I think, I would kick them out or something like that. For, then it wouldn’t work anymore. […] I think I imagine things that never existed or were there […], that they talk about me, that they don’t need me, that I do them no good, or something like that.

In relation to the second hallmark (a background sense of being upset either expressed as aggressiveness or transformed into numbness), both participant #2 and participant #6 described a pattern of intermittent dysphoric states of at least three different kinds: an expansive mood, repetitive episodes of a profoundly sad affect, and phases in which they felt affectively empty and almost dissociated. Particularly, the narrative of participant #6 conveyed the impression that this expansive mood situationally expressed a background sense of anger, which her sadness subsequently transformed in such a way that a phase of emotional bluntness was afterward able to conceal her profound rage:

[…] I mean this … that I fall into this black hole at school and then stay all day there, that I’m mega-aggressive for no reason, so really nothing happened and I’m so aggressive that you can’t talk to me anymore. Then I’m sad for no reason, then I just cry, even though nothing has happened. That is …, I don’t know, right now it is …, and then … either, when I’m freaked out, then I fell into this black hole and then it’s completely “radio silence,” then you can tell me what you want, then I don’t have anything more, then I just don’t feel anything more, luckily I’m no longer aggressive, but that’s, for instance, in school the case that I need a protection-shield, too, such a way that no one notices that I feel bad, I then pretend that I am fine.

This participant seemed to experience her “radio-silence” phases as involving a sort of mechanism that protected her from both acting out her anger in an aggressive way and letting others recognize that she was actually not fine.

Concerning the third hallmark of the Upset (Refusing) Mode (extreme dependence of mood on feelings of social rejection), participant #2 described this idea when mentioning that her impression of having been insulted or dismissed by a significant person usually led her to suffer the mood alterations that she associated with depressive episodes: “Well, most of the time when I have to cry, I’m hurt because my ex-boyfriend insulted me again or told me to go die.” For her part, participant #6 described how, after getting the unbearable impression that other people regarded her as a burden, she closed herself off to any social interaction:

[…] I don’t want to talk to anyone and I can’t talk to anyone, either. I think …, I don’t know, it’s just always like that, it’s like that. No feeling there, but there are just thoughts there. So, now not suicidal thoughts, but such thoughts like, “Yes …, you were a burden today, you were a burden today, you were a burden today,” like this.

The point is not merely that during these episodes adolescents shunned social interaction. What is unique is their uncompromising resistance to offers of social contact. These adolescents conveyed how, in the relevant phases, they did not allow interpersonal exchanges to occur, even though they felt they needed them. It is particularly interesting that, on participant #6’s account, this radical and paradoxical social closeness was regarded as a sort of defiant refusal of attempts to “calm” her sorrow: “So, I do not know, if you are sad, depressed …, I do not like it, for example, when someone wants to hug me or help me, I just want to keep being depressed or sad.”

Moreover, clarifying that it was not her relation to adults (as opposed to her relation to peers) that became transformed, but her relation to important adults (as opposed to her relation to persons with lesser relational relevance to her), participant #6 said,

It all depends on what kind of adults they are. Even with my mom, for example, I also have […] this feeling that she doesn’t need me or that I’m a burden and so on … Yes, but, I don’t know, with others I’ve never thought about it like that, I don’t care either.

Thus, the described transformations seem to concern specifically the connection to people these participants care about or deem important—people they are attached to. We believe that this is characteristic of this group.

Finally, two accounts informed our characterization of the Paralyzed (Non-Resonating) Mode, namely those of participant #1 and participant #9. These accounts bring to the fore a feeling of meaninglessness experienced with respect to a life that these participants felt should “somehow keep on going anyway.” This is related to a sense of deep apathy, exhaustion, and inability to change their situation.

The first hallmark of this mode of depressive experience concerns the kind of generalized resignation that we captured under the description of a “tragic tolerance of an existence that seems to have no point.” Participant #9, for instance, conveyed a sense that, in existing, she was merely attempting to, in some way, deal with or circumvent (“get around”) a life that does not merit being lived: “Yes, life [is experienced] definitely different … just as unbearable, as negative, as often not necessarily worth living, I would say …, it seems too long, and, yes, you do not know how to get around, what to do with it.” At certain moments, this acceptant resignation included a sort of identification with aspects of the condition as emerging from the affected adolescent’s character. Concerning how she initially thought about her focus on negative things, participant #1, for instance, said, “It’s just because of my personality that I think about things like that.”

The second hallmark we identified for the Paralyzed (Non-Resonating) Mode concerns the markedly anhedonic state that these adolescents stressed. As participant #9 put it, there is “a lot of demotivation, a lot of indifference to everything.” Ultimately, this was experienced as a deep apathy, i.e., as a feeling of being unresponsive to “possibilities” recognized as worth being actualized but not experienced as motivating.

This feeling was accompanied by a sense of profound cognitive and motoric limitation. Participant #9 said, “Yes, you cannot really grasp thoughts.” In relation to her episodically transformed sense of embodied agency, she expressed later, “And depending on the phase, it may also be that you still somehow perceive your body, but you do not …, how should we put it, [you] do not feel in the sense …, well, you cannot move, […] in the truest sense of the word, not a finger anymore.”

These elements converge in the struggling experience of powerlessness that we characterized as a state of pseudo-immobility, which participant #9 articulated as follows: “[Y]ou’re kind of incapacitated and incapable of helping yourself. But the fetters do not come from without, they come from within.” For her part, participant #1 said, “[I]n these moments, I do not think, ‘Well, I cannot get up now, because it does not make sense anyway.’ It’s just the way it is. It feels like it just cannot be done.”

Thus, this mode reflects the sort of incapability experienced by a person who can no longer understand herself as an agent, despite knowing that she possesses physical capacities to move and transform. Differentiating this condition from a “genuine” state of paralysis, participant #9 said, “When you are in the state of ‘being-fettered,’ I would say, then … you still track your body and you know that you could theoretically move, but you cannot, I would say, and in the other case you simply cannot.”

### 3.3 The phenomenological specifiers in relation to trajectories of personality development

**Figure 1:**
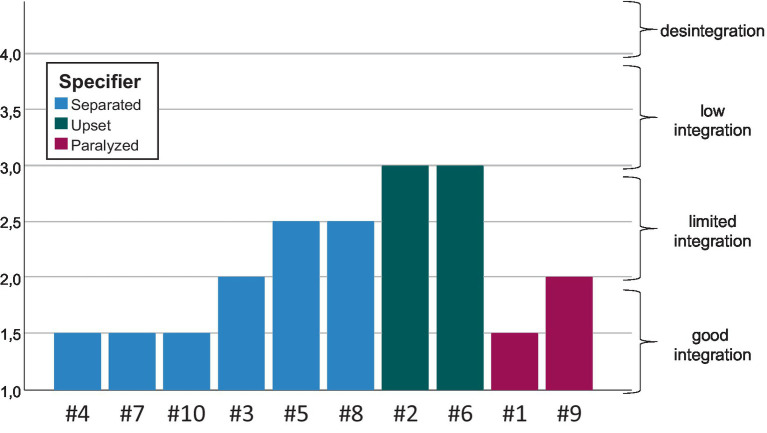
The specifiers in relation to the structural integration level (OPD-CA-2).

**Figure 2:**
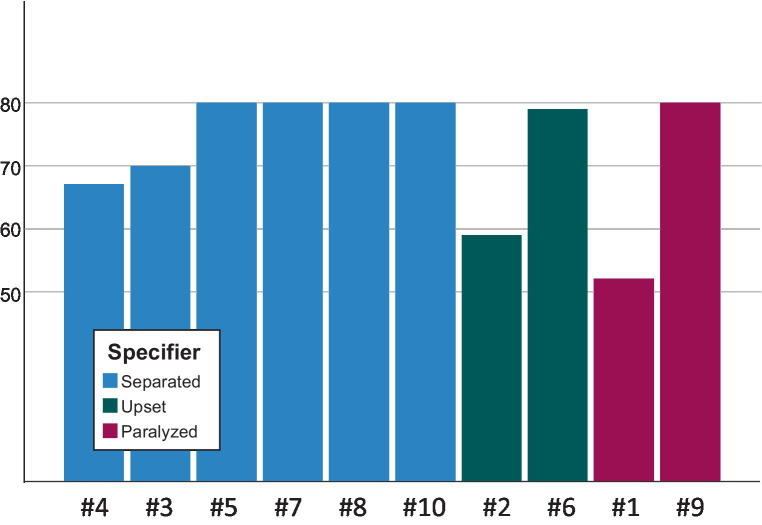
The specifiers in relation to the scale “Identity Diffusion” (AIDA).

In the last step of the study, we put the emerging specifiers in relation to our results of the assessment of the participants’ personality structure (OPD-CA-2) and identity development (AIDA). The following two figures summarize the results (Figures 1, 2). To facilitate comparison, we reordered the participants within the graphs.

We non-statistically identified an association between the specifiers and our participants’ personality structures. This correspondence suggests that on the basis of our classification of the varieties of adolescent depression, it is somewhat possible to “anticipate” results of a structural integration assessment. Particularly, both participants whose accounts were classified under the Upset (Refusing) Mode specifier exhibited a higher OPD-CA-2 score, indicating a less well-integrated personality structure that points to a (higher risk of developing a) personality disorder (Figure 1).

Against the background of an influential claim, according to which the syndrome of identity diffusion would represent the core of personality disorders (cf. Clarkin et al., 1999), one might expect the achieved phenomenological classification to also be associated with the total score “identity diffusion” of the AIDA questionnaire. However, we could not confirm this association (Figure 2).

## 4 Discussion

### 4.1 Thematic convergences and comparability of our results with those of other qualitative studies

This study investigated the ways in which young people describe their relation to the world and to themselves when they are going through a depressive episode. Specifically, it examined our intuition that thematic motifs that appear central to personal accounts of depression may recur in a distinctive manner across narratives of affected adolescents—in a way that allows for proposing a classification. Despite our emphasis on varieties of adolescent depression, we first must note that there is significant thematic overlap across the differentiated experiential modes.

Here, these convergences are not a problem. As mentioned above, the proposed characterizations do not aspire to define mutually exclusive phenomenological subgroups that jointly exhaust the diversity of experiences associated with the diagnostic category “depression.” This is why, following a terminology well established in DSM-5, we talk of specifiers (in the sense of features that may differentially indicate specific clinical interventions in certain patients who have received a particular diagnosis).

Further, the partial overlap may have arisen because all the analyzed personal accounts describe conditions that can be grouped under the category “depressive episode.”^4^ In fact, given these convergences, one might want to validate our results by comparing them to those of other qualitative studies that have aimed to capture the lived experience of adolescents diagnosed with depression but were not primarily interested in differentiating phenomenological varieties. However, such a comparative external validation is limited by our understanding of what a phenomenological-psychopathological exploration amounts to.

For example, some of the motifs of adolescent depression mentioned by our participants certainly reflect the topics identified by other qualitative studies, like the following themes reported by Crowe et al. (2006): feeling fatigued, being unable to concentrate, feeling slowed down, being unable to hope, feeling helpless, and feeling compelled to engage in self-harming activities. Relatedly, some of the themes of adolescent depression identified by other authors who conducted in-depth interview studies (Farmer, 2002; Midgley et al., 2015; Weitkamp et al., 2016; cf. the introductory discussion above) are “proximal” to the motifs we described for the first-rank thematic bundles (cf. Supplementary material). However, it is not clear how to assess the profundity of such correspondence claims, specifically because the term *phenomenology* can have different meanings in philosophical, scientific, and clinical contexts.

Even for psychopathological investigations that claim allegiance to Husserl or other figures of the phenomenological tradition, we are cautious not to assume that their goal was to reveal what we, drawing on the philosophical considerations discussed above, are calling the *phenomenological centrality* of a motif. Indeed, Berrios argued that “Jaspers’ [canonical] contribution to descriptive psychopathology (DP) is, in the main, independent of the philosophical movement called ‘phenomenology’” (Berrios, 1992, p. 303).

Moreover, some of the outcomes of our interpretative analyses may seem to contrast with the results of other studies. For example, based on our findings, the widely shared view that irritability experientially defines adolescent depression (cf. Crowe et al., 2006) should not be endorsed, although anger was mentioned in some of the accounts we analyzed. Similarly, the issue of poor-quality sleep, which has been found to constitute a common symptom of adolescent depression (cf. Lachal et al., 2012), did not define the experiences of our participants, although this symptom was also reported in our sample. These and other apparent discrepancies point to a crucial issue that we must underscore: Neither the frequency nor the intensity of a symptom can in itself determine the phenomenological centrality of any motif.^5^ For this reason, without a more in-depth meta-methodological discussion—which would definitively exceed the scope of this article—we cannot equate the predominance of a specific topic reported by another qualitative study with the phenomenological centrality we investigated.

Despite this possibly restricted comparability of findings, we nonetheless discuss our results in the context of extant qualitative and philosophical-phenomenological studies on experiences of (adolescent) depression. This seems warranted, since the results of these studies may suggest revisions to the current view of (adolescent) depression that converge with some of our points. For instance, challenging the popularity of claims concerning the significance of irritability in adolescent depression, a population-based survey reported that, “[d]epressed mood was the most common cardinal mood in youth meeting criteria for depression” (Stringaris et al., 2013, p. 831). It is important to note, though, that, in the context of a properly phenomenological exploration, it is insufficient to point to a series of “brute facts” concerning the experiences of affected adolescents. If we are to understand phenomenologically what we call *experiences of adolescent depression*, then we must specify what it is like for an adolescent to be in such a “depressed mood.” Particularly, we must spell out the sense in which this may be grounded in a transformation of certain structures of relatedness.

### 4.2 What it is like to be in a “depressed mood” at a young age

Our participants often reported a painful sense of interpersonal disconnectedness and distance from the world. Almost every idiographic analysis delivered a thematic bundle associated with this topic. This is concordant with studies that found a sense of loneliness (Lachal et al., 2012) and withdrawal (McCann et al., 2012), a feeling of “emotional homelessness” (Farmer, 2002), and a sense of isolation (Midgley et al., 2015) to be predominant in reported experiences of adolescent depression. These findings converge with philosophical-phenomenological analyses of accounts of affected adults that emphasize a depressive feeling of disconnection (Osler, 2022) and a sense of not being at home in the world (Ratcliffe, 2015). Based on our analyses, we try to phenomenologically characterize this sense of having been detached from the (social) world in such a way as to point to a “typically” depressive modification of structures of experience.

Midgley and coauthors highlighted an issue that may allow us to begin spelling out this particular feeling of disconnectedness: the felt “lack of emotional understanding” (Midgley et al., 2015, p. 275). A related impression of being fundamentally—as opposed to situationally—misunderstood was very common across the personal accounts we analyzed. This topic was relatively highly ranked in the thematic bundles of most participants who mentioned it. One of our participants put this sense of “misunderstandingness” in the center of her account and employed this expression as a label apt to capture the overall way of being-in-the-world for an adolescent with depression (cf. Sánchez Guerrero, 2023). This is concordant with findings of other interview studies (cf. Dundon, 2006).

Moreover, Osler (2022) suggested that this feeling of being misunderstood could be conceived as the *cause* of the sense of disconnection that she centrally associated with the phenomenology of depression. In line with this suggestion, a topic mentioned by Midgley and coauthors may further illuminate the particular feeling mentioned by our participants of having been cut off: “the sense of confusion that many young people describe in relation to their depression” (Midgley et al., 2015, p. 271). As reported, in our sample many adolescents described a painful sense that their inner world had become ambiguous or even paradoxical and incomprehensible, and this sense constituted an important element of the depressive predicament. This feeling sometimes made the affected adolescents’ external world appear chaotic, foreign, and even estranged.

It might be objected that such a sense of confusion apt to transform the life-world is unspecific with regard to psychopathological experiences of different kinds. For instance, Louis Sass (1992) argued that an all-embracing irony associated with a hypertrophied self-consciousness, leading to a sense of existential puzzlement, is at the core of schizophrenic being-in-the-world. However, our analyses suggested that a *particular* sort of experienced contradiction that is integrated in how affected young people find themselves in the world constitutes a key phenomenological aspect of adolescent depression. Moreover, as mentioned, the three varieties of “depressed attunement to the world” specified above (Separated [Yearning] Mode, Upset [Refusing] Mode, and Paralyzed [Non-Resonating] Mode) may capture the distinct “existential incongruity” that is proper to each of the distilled modes of adolescent depression.

We propose that, coming in different varieties, a specific kind of felt paradoxes determines possible ways in which depressed young people can experientially relate to the world and to themselves. Put another way, we believe that a series of themes that are central to experiences of adolescent depression all express a specific sense of (self-)discrepancy associated with a morbid modification of structures of relatedness. In its most generic form, this felt incongruity concerns a painful sense of somehow being a part of the world, but, at the same time, not really being able to participate in most worldly occurrences—a sense of being apart from the “habitual world.” Such a Janus-faced background experience—a paradoxical form of (dis-)connection—seems to be phenomenologically so determining that the specification of forms of depressive experience in adolescence may be articulated in terms of *modes of being a-part*. [A philosophical elaboration on this suggestion will be offered elsewhere (Sánchez Guerrero, in preparation).]

### 4.3 The identified phenomenological varieties in relation to proposed types of depression

Our specification of three varieties of adolescent depression does not correspond to any of the classifications proposed so far in the literature. However, our phenomenological specifiers seem to coincide with aspects of types of depression that have been differentiated elsewhere.

We proposed above that diverse components of the Paralyzed (Non-Resonating) Mode converge on a throbbing experience of ineffectiveness and pseudo-immobility. Such a sense of powerlessness has been argued to explain—as opposed to being derivable from—the depressive mood of patients with melancholic depression (cf. Ballet, 1911; as referred to by Shorter, 2007). Specifically, the negative mood of individuals affected by melancholic depression might be secondary to a sense of loss of energy and interest in everyday activities. This is relevant to our discussion, since, as mentioned, apathy centrally determines the accounts provided by participants #1 and #9 and, particularly, their feeling of incommunicable exhaustion. However, this is definitively not sufficient to equate the mode of depressive experience captured by the Paralyzed (Non-Resonating) Mode specifier with “melancholic adolescent depression.” Particularly, we cannot clearly attest that somatic symptoms are the distinct phenomenological fundament of the psychopathological experiences at issue.

In our view, the question concerning this potential relationship is completely open. The descriptions of both participants whose accounts we grouped under this specifier sound much like the “weariness of the self” sociologically distilled by Ehrenberg (1998/2010). However, we cannot exclude the possibility that effects of psychomotor and thought retardation associated with an endogenous component of the disorder may *bring* certain individuals to experience such a “fatigue d’être soi” at a very early age.^6^

In relation to the Separated (Yearning) Mode, we first must point to the fact that it applies to the descriptions of most of our participants. This could lead to the impression that this first specifier represents the most common form of adolescent depression. However, an alternative interpretation is that among the specifiers worked out, this one is the most unspecific. Particularly, at the core of this ideal-typical characterization is something that defined the experiences of practically all our participants: a persistent sense that they had been detached from the world. In fact, this altered background experience—this sense of no longer being immersed in a world they take themselves to be a part of—was so pervasive that, as discussed, we characterized the phenomenological core of adolescent depression *tout court* in terms of a sense of being a-part.

Yet, we pinpointed two features that distinguish this form of depressive experience. The first concerns a sort of anti-identification with the morbid condition, which was less central to accounts grouped under the other two specifiers. The second feature concerns the relatively selective character of the sense of disconnection to the social world. Participants whose accounts were classified under this specifier described a distance to peers, whom they often experience as hostile, but a relatively intact access to the support, companionship, and/or kindness of their caregivers.

We believe that both highlighted phenomenological aspects point to something fundamental that unites this subgroup of adolescents diagnosed with depression: these participants displayed a relative integrity concerning their attachment representations. Against this background, we can begin appreciating the importance of recognizing the sort of predicament captured by our second specifier.

The Upset (Refusing) Mode of adolescent depression had three specific hallmarks, one of which was the experience of having evolved a potentially destructive attitudinal rage (which we suggested to be related to a diffuse feeling of having been offended by important others). This is concordant with findings reported by Midgley and coauthors, according to which “[f]or *some* [adolescents], the feeling of anger was what they meant by ‘depression’” (Midgley et al., 2015, p. 274, our emphasis). The authors were eager to stress that their emphasis on anger goes beyond the usual claim that irritability is as a symptom equivalent to depressed mood in adolescent depression (cf. Midgley et al., 2015, p. 276–277). The form of anger they pointed to—which usually involves self- or other-directed violence—should, thus, be regarded as a feature of a certain mode of depression in adolescence, not as a hallmark of adolescent depression generally.

Both participants whose accounts were classified under the Upset (Refusing) Mode specifier had been diagnosed with a mixed condition including symptoms of a disruptive disorder and symptoms of a depressive episode (see Table 1). Comorbidity could, thus, explain the fact that anger and aggressive impulses played such an important role in their characterization of the depressive predicament. However, comorbidity (a disruptive disorder plus a depressive disorder) may not best explain the mentioned phenomenology. An alternative view could be that a single condition—a unitary transformation of the experiential field—may involve (1) symptoms that allow diagnosing a depressive episode and (2) recurrent experiences that turn on the feeling of having been offended in some way. The latter experiences may, in turn, lead to (3) forms of comportment that are classifiable as a disorder in the domain of social behavior. Indeed, adolescents with strong depressive symptoms have been shown to be at increased risk of disruptive behavior problems (cf. Wolff and Ollendick, 2006; Reinke et al., 2012). In other words, these allegedly distinct domains of comorbid psychopathology may be interconnected by means of a single process in such an intricate way that the diverse symptoms should be understood as aspects of the same disorder. Supporting this view—which seems to be implicit in the ICD-10 notion of such a mixed condition—would require being able to empirically track the distinction between episodic and chronic irritability. Our design does not allow us to do so.

Another hallmark of the two accounts we grouped under the Upset (Refusing) Mode specifier is that they both conveyed a particular susceptibility to interpersonal rejection that led to a contradictory closure to the social world on which the affected adolescents actually felt to be dependent. Participants whose accounts were classified under the other two specifiers also described a tendency to social withdrawal and isolation that might appear paradoxical, given their felt urgency to engage in potentially comforting social interaction. However, for the Upset (Refusing) Mode, participants showed an extreme sensibility to experienced interpersonal dismissal, and on certain occasions such dismissal caused them to completely refuse social encounters and even make suicidal depressive crises; thus, we considered this extreme response as a hallmark of this form of adolescent depression.

Liebowitz and Klein (1979)—who consider that an extreme intolerance to interpersonal rejection constitutes the central feature of the disorder they call *hysteroid dysphoria*—argued that a particular vulnerability to loss of romantic attachment is characteristic of this dysthymic condition. Participant #2, in particular, expressed that her depressive phases depended on disturbances within her relationship to her boyfriend. However, we believe that the vulnerability at issue concerns attachment as a general dimension of personality.

In this context, we find it particularly interesting that, as reported, both participants whose accounts were classified under the Upset (Refusing) Mode specifier described transformations of their relationship to caregivers during depressive episodes. As mentioned, these series of transformations, which seemed to specifically concern relationships with people to whom they were attached, could be regarded as characteristic of this kind of depressive experience. Notably, participants whose accounts were classified under the other two specifiers described either no specific alteration or a modified relationship to peers who appeared more distant during the depressive episode; relationships to their caregivers tended to remain unchanged.

The notion of “hysteroid dysphoria” is highly controversial, so we refrain from moving the discussion in that direction. However, we believe that the emerging impression concerning a particular sensitivity to interpersonal rejection that preferentially concerns persons of import points to an interesting connection between the Upset (Refusing) Mode and experiences within the context of (early) caregiving relationships. In the next subsection, we discuss a general issue related to this connection: the dependence of the reported depressive experiences on adolescents’ developing personality structure.

### 4.4 Varieties of adolescent depression and the developing personality structure

Our design was informed by a series of findings that suggest a relationship between certain kinds of experiences of depression and the comorbid presence of a borderline personality disorder (cf. Westen et al., 1992; Wixom et al., 1993; Blatt, 2004; Levy et al., 2007; Silk, 2010; Köhling et al., 2015). We preferred to frame the issue in terms of a possible dependence of the phenomenology of adolescent depression on the individual’s personality development trajectory (cf. Sánchez Guerrero, 2023). Our reason for doing so was twofold.

First, in child and adolescent psychiatric practice, a resistance remains to diagnose personality disorders. Therefore, if one is to study a possible relationship between developing character pathology and phenomenological varieties of adolescent depression, it seems reasonable to avoid categorical discriminations concerning the presence of a full-fledged personality disorder. Accordingly, we put the results of our phenomenological analysis in relation to a continuum of personality development. As mentioned, we articulated this continuum along a dimension of levels of integration of the personality structure. This is in accordance with the way in which both ICD-11 and the Alternative Model for Personality Disorders in DSM-5 (Section III) conceive of personality disorders by focusing on dimensions of core personality exhibiting maladaptive functioning.^7^

Second, through this study we aimed at inaugurating a trans-methodological field of inquiry pertinent to child and adolescent psychiatric theorizing. This field has been set to establish a bridge between phenomenological and developmental psychopathology. In this context, we found Blatt’s (2004) seminal work on experiences of depression particularly inspiring. Blatt distinguished experiences of two kinds of depression: first, the interpersonally oriented (anaclitic) kind, which is based on feelings of loneliness and loss, and, second, the introjective kind, which turns on feelings of low self-worth. Blatt made a case for the idea that distinct lines of development of a person’s representation of caregiving relationships—which constitutes a central dimension of the personality structure—determine a differential vulnerability to the distinguished varieties of experiences of depression.^8^

An incidental finding of our study (not reported above) reinforces this idea that different lines of psychopathological development related to early caregiving relationship experiences could (partially) account for the identified phenomenological varieties of depression. This finding pertains to the relatively limited capacity of both participants whose accounts were classified under the Upset (Refusing) Mode specifier to articulate in words their own experiential life (cf. Supplementary material, Section B).^9^ Here, their limited ability to do so may relate to general deficits in these participants’ capacities to understand human behavior in terms of intentional attitudes. As theorized, the development of the ability to mentalize depends on the quality of certain interactions between an infant and her primary caregivers (cf. Fonagy et al., 2002). Thus, a link might exist between this functional psychological limitation and the phenomenological centrality of themes concerning a sensibility to rejection by the part of persons of import in the accounts grouped under the Upset (Refusing) Mode specifier.

The capacity to mentalize and a series of related abilities (e.g., the capacity to empathize and to engage in reciprocal emotional exchanges) are not the only developmental capabilities that depend on the quality of early caregiving relationships. Many further capabilities that constitute the personality structure (e.g., the ability to regulate affects or to adequately “use” attachment figures and/or representations) also exhibit this dependence. Accordingly, this incidental finding might reinforce the expectation of a relationship between the worked-out phenomenological varieties of adolescent depression and the trajectory of personality development.

As reported above, only some of our findings confirmed this expectation, since the assessment of our participants’ identity development by means of the AIDA questionnaire provided results that did not map to our specified phenomenological discriminations. The reason we could not confirm this expectation may be related to factors that cause two instruments (OPD-CA-2 and AIDA) that are informed by the psychodynamic tradition of thought and both operate with the notion of identity diffusion to exhibit this surprisingly poor correspondence. In our view, some of these factors might be related to the different nature of these instruments, thus, understanding these instruments’ respective goals may deliver *prima facie* reasons for taking seriously the relationship between the Upset (Refusing) Mode specifier and a low-level structural integration as assessed by the OPD-CA-2, despite the missing expected concordance across instruments. Yet, a discussion of these factors would distract from the main message of this contribution and, thus, should be carried out elsewhere.

### 4.5 Possible implications for clinical practice and future research directions

Our findings suggest an association between different sorts of experiences described by adolescents diagnosed with depression and the integration level of their personality structure. Thus, certain “themes of adolescent depression” might reflect a variable related to a developmental goal that is fundamental to consolidating a healthy character: the goal of establishing a consistent sense of selfhood that allows meaningful spaces of relatedness and coherent expressions of autonomy. On theoretical grounds, such a variable might be assumed to summarize how a series of early life determinants related to the organization of internal working models and self-regulative capabilities influence personality development (cf. Fonagy et al., 2002). For this reason, it might be enormously clinically relevant to confirm the proposed association in future (hypothesis-testing) empirical studies. In what follows, we specify this clinical relevance and suggest future research directions.

Given the modest evidence in favor of antidepressants in the pediatric population (cf. Wagner and Ambrosini, 2001; Kennard et al., 2006; Bridge et al., 2007; Vitiello and Ordóñez, 2016), most cases of adolescent depression are treated via psychotherapy. In the psychotherapeutic context, creating interventions personalized to the patient’s experiential profile could lead to more specific and contextually more effective therapeutic measures. Therefore, the phenomenological specification of varieties of depression could be particularly pertinent in child and adolescent psychiatry.

Assessing an adolescent’s personality development is time-consuming and implies the use of sophisticated diagnostic instruments. Such an assessment is rarely included in the diagnostic evaluation of an adolescent complaining of symptoms typical of depression. On the other hand, without obtaining descriptions of how affected adolescents experience themselves and their world during relevant episodes, no current clinical assessment can reveal a depressive disorder at a young age. Thus, over a few clinical encounters, a practitioner sensitive to the specifiers we worked out might easily recognize the importance of certain descriptive motifs in an adolescent’s account of her dysthymic condition. In this context, the centrality of themes related to the Upset (Refusing) Mode could be used to indicate that certain adolescent patients consulting mental health services with symptoms of depression should have their personality development assessed. Moreover, if future studies can strengthen this association between the Upset (Refusing) Mode and (potential) personality disorders, an adolescent diagnosed with depression that predominantly describes themes cardinal to this phenomenological specifier could indicate to clinicians that they should employ therapeutic interventions designed to treat deficits pertaining to the personality structure (e.g., restricted mentalizing capabilities, insufficient and/or dysfunctional emotion regulation strategies, identity diffusion).

Further empirical and philosophical studies could examine the plausibility of our phenomenological proposal that changes to adolescents’ relational space are associated with what we call *feelings of being a-part*. These examinations might assess the extent to which such a sense that contradictory aspects constrain one’s habitual experiential life is (1) frequently encountered in cases of adolescent depression; (2) characteristic of those forms of pathology that can be classified as a depressive disorder; and (3) persistent across the life span, despite the heterotypic continuity often argued to be typical of depressive symptoms.

Finally, German and British practice guidelines consider pharmacotherapy predominantly in cases of adolescent depression for which no sufficient response to psychotherapeutic interventions was seen. The specification of varieties of adolescent depression we worked out does not suggest instances in which medication should be tried earlier. However, as suggested, further studies may explore the relationship between certain sorts of experiences of adolescent depression—particularly those characteristic of the Paralyzed (Non-Resonating) Mode—and the kinds of dysthymic disorders traditionally associated with melancholic endogeneity, which apparently respond better to biological therapeutic measures.

### 4.6 Limitations of the study

The most important limitation of this study relates to generalizing our results to the relevant population. Qualitative inquiries based on idiographic interpretations of in-depth interview transcripts typically involve extensive (and time-consuming) analyses of material obtained from conversations with relatively few participants. While the data from these studies is typically not scarce, it is definitely problematic to draw generalizable conclusions from a small number of cases to a population that exceeds the relevant sample.

However, to suggest that a phenomenologically oriented interview study with a typical small sample size is, in principle, insufficient to support generalizations is to assume that all scientifically relevant generalizations are based on inductive hypothesis-testing experiments. Yet, deductive generalizations that require zero cases may be crucial for processes that generate a solid scientific view of some phenomenon. Further, generalizations of a theoretical sort are achieved by contextualizing findings in an edifice of received knowledge—knowledge that has typically been gained via a mixture of inductive and deductive processes. As argued elsewhere (cf. Sánchez Guerrero, 2023), phenomenological-psychopathological generalizations that might, in principle, even draw upon the analysis of a single case are typically of this latter kind.

Despite this distinction, in the context of our empirical exploration of the varieties of adolescent depression, the following fact could still appear problematic: Given our point of departure—the impression that experiences of adolescent depression are highly variable—it is probable that a larger sample could have led to a different result. For instance, analyzing interview transcripts of 50 additional depressive adolescents could have suggested a different phenomenological typology. So, should we have designed the study with a larger sample?

Similarly, we could have asked whether we should have controlled thinkable confounding factors. For instance, given that the content of the analyzed narrative material is at least indirectly related to the quality and degree of differentiation of the accounts, controlling participants’ cognitive and verbal capacities could have been important for identifying certain potential factors that may have led to alterative explanations of the results. Moreover, as mentioned above, many of the various “themes of adolescent depression” distilled in different studies could also have been expected to arise in the personal accounts of adolescents diagnosed with other psychiatric conditions—or even with no disorder whatsoever. As such, we perhaps should have included a control group of adolescents that had not been diagnosed with depression.

However, to require that explorative studies meet the standards of rigor for hypothesis-testing studies is to misunderstand the specific function of explorative investigations: to begin illuminating a relatively virgin field of knowledge that may not even have clear terminology yet to use for posing empirical questions. Indeed, we can conceive of an exploratory study design that aims to achieve a particular sort of generalizations; namely, the sort of *preliminary* generalizations that are formulated precisely to generate plausible categories that may eventually lead to empirically testable hypotheses.

In the present study, the generalizability issue is complicated by the fact that we are proposing an association between two types of results: the emerging phenomenological specifiers, on the one hand, and the results of our assessment of the personality structure, on the other. To demonstrate that a presumable association is not just the product of mere chance requires an inductive design with an adequately powered sample, which, depending on the impact of the relationship at issue, should be much larger. Moreover, to allow replications, such a design should specify the statistic computations that permitted recognizing the relevant relationship. However, we drew our conclusions concerning a possible association from a sheer tabular juxtaposition (i.e., a non-statistical pairing) of the two relevant sets of findings.

Our rationale for doing so is again related to the pre-concept nature of this inquiry. Particularly, our goal was to investigate a relationship that after the study might (or might not) *contingently* appear more probable, in the sense of appearing not merely theoretically plausible (as this was somewhat already the case before the study) but also illustratable in an economically chosen sample—as opposed to demonstrable in an adequately powered sample.

From the beginning it was obvious that our sample was, although relatively large for a qualitative study based on idiographic analyses of in-depth interviews, too small for detecting significant differences not recognizable via sheer juxtaposition of the relevant relata. While designing the study, we did not merely accept this consequence of our small sample; rather, we decided to restrict any potential claim concerning a possible association between the relevant results to differences that may be detected without statistical magnification. As we conceived, given the complete uncertainty of the empirical basis of any claim regarding the possible relationship we worked out, only a maximally evident indicator of such an association could immediately justify the efforts and costs associated with an adequately powered future hypothesis-testing investigation.

Another limitation of this study was that we did not use an assessment of depressive symptoms involving reliable severity ratings based on structured diagnostic interviews, as one may argue that the severity of depressive symptoms could play a role in determining the specific phenomenology of a case of adolescent depression. For instance, Crowe et al. (2006) suggested that irritability as a key feature of adolescent depression depends on the severity of depressive symptoms. However, we believe that these claims of association merely correlate a general symptom score with the presence of a certain specific symptom; they do not really point to an association between severity of the symptomatic and a specific transformation of structures of experience. Our decision to forego time-consuming assessments of symptom severity was, thus, consistent with our conviction that a depressive condition exhibiting several symptoms—the basis on which severity is usually determined—does not necessarily lead to more radical transformations of the experiential field. In fact, this does not even necessarily lead to a more incapacitating disorder (cf. Gotlib et al., 1995; Solomon et al., 2001; Fried and Nesse, 2015).

A further limitation of our study may be related to our patients having been referred to a specialized mental health service (to a university hospital). As such, our participants made up a sample that probably does not represent the group of adolescents meeting criteria for a depressive episode in the general population.

Additionally, our recruitment relied on diagnostic criteria that are slightly different than those likely to guide future studies (the criteria of the DSM-5 or ICD-11), which could have further implications concerning the restricted validity of our results.

We did not systematically differentiate unipolar from bipolar depression. This could appear relevant in larger studies.

These limitations certainly constrain our findings regarding their generalizability and specificity. However, in our view, they do so to an extent that is reasonable for a study with the ultimate practical goal of proposing some terms upon which a hypothesis-testing exploration can be designed to empirically track an association that has been vaguely suggested by the pertinent literature.

## 5 Conclusion

This study examined the intuition that thematic motifs central to personal accounts of depression may recur in a distinctive way across narratives of affected adolescents. Our impetus was that such a differential recurrence might permit classifying accounts of adolescent depression according to their core themes. This, in turn, could allow for specifying varieties of adolescent depression in a phenomenological way.

At the methodological level, this qualitative study explored the possibility of gaining insights relevant to child and adolescent psychiatry by employing a systematic of interpretation derived from a mode of thinking that aims to reveal conditions of intelligibility of reported changes in the experiential world. In this way, our investigation tested the power of an approach that—despite its long tradition in adult psychiatry—has failed to permeate child and adolescent psychiatric research: the phenomenological-psychopathological approach.

The Depression Experiences Interview—an interview setting designed for the purpose of this investigation—permitted us to obtain descriptive material that was rich enough to generate categories that may eventually lead to empirically testable hypotheses. Our devised interpretative procedure proved appropriate to distill experiential field modifications associated with psychopathology in adolescence. This allowed us to extend a series of first impressions arising from the few qualitative analyses of adolescent depression published up to date. In combination with our assessment of the participants’ personality structure, this phenomenological investigation reinforced the idea that the notorious heterogeneity among experiences of adolescent depression could be classified in ways that have potential therapeutic significance. Hence, our findings allowed us to propose the first phenomenologically grounded specification of varieties of adolescent depression with potential clinical relevance.

Based on our results, we suggest an association between the specified varieties of adolescent depression and the adolescent’s personality development trajectory. In the face of existing psychotherapeutic interventions that enable targeting specific deficits of the personality structure, the confirmation of this association might have enormous therapeutic relevance. Further studies are certainly required to assess whether the proposed association is sufficiently robust. However, we believe that this contribution makes an opening case for the idea that modes of scientific exploration thematically and methodologically informed by phenomenological philosophy could offer a foundation for theorizing in the context of child and adolescent psychiatry.

## Data availability statement

The datasets presented in this article are not readily available because ethical issues related to the possibility of personally identifying participants constrain the possibility of making complete interview transcripts publicly available. In the Supplementary material, readers can find results of intermediate analytic steps. This is expected to permit evaluation of the plausibility of relatively abstract or generalizing claims made in the article. Requests to access the datasets should be directed to HectorAndres.SanchezGuerrero@ukmuenster.de.

## Ethics statement

The studies involving humans were approved by the Ethics Committee of the Medical Council Westfalen-Lippe. The studies were conducted in accordance with the local legislation and institutional requirements. Written informed consent for participation in this study was provided by the participants’ legal guardians/next of kin. Written informed consent was obtained from the minor(s)’ legal guardian/next of kin for the publication of any potentially identifiable images or data included in this article.

## Author contributions

HASG: Conceptualization, Investigation, Formal analysis (qualitative analysis of the interview transcripts), Methodology, Project administration, Writing – original draft, Writing – review & editing. IW: Formal analysis (rating of the OPD-CA-2 interviews), Visualization, Writing – review & editing.

## Footnotes

^1^In this contribution, we employ the adjective *dysthymic* to characterize generally a diversity of clinically relevant conditions associated with a dysphoric mood. Thus, here we do not mean the specific diagnostic category *dysthymia*.

^2^To minimize the possibility of personally identifying our participants, we are deliberately omitting individual information concerning their gender and age. Correspondingly, in the remainder (and in the Supplementary material) we shall report about personal participants’ experiences in a strictly anonymous way, indistinctly employing female pronouns and, if required, slightly modifying the content accordingly.

^3^One of us (H.A.S.G) translated the original quotes from German into English. In the Supplementary material (Section A), readers can find further results of intermediate analytic steps. Specifically, we present the hierarchies of thematic bundles that emerged during our idiographic analyses of the DEI transcripts. For this contribution, the statements emerging from our analyses have also been translated from German into English (by H.A.S.G). This is expected to permit evaluation of the plausibility of our suggestions. Due to ethical issues related to the possibility of personally identifying participants, the DEI transcripts have not been made publicly available.

^4^Our approach to considering narratives as valid accounts of depression was typically clinical: as mentioned above, our participants were included based on symptoms that formed some constellation sufficient to fulfill criteria for diagnosing a depressive episode. For this reason, we do not discuss whether the proposed specifiers should be taken to discriminate *different disorders* that are misleadingly categorized together or *diverging expressions* of a legitimate psychopathological entity. In our view, treating the diverse predicaments described by our participants as “cases of depression” is in accordance with current clinical practice. Moreover, at the moment, this is probably the most parsimonious way to connect to extant research—which is normally conducted at the level of the major depression domain—while making room for discussions about whether the mainstream scientific discourse on depression “effectively swamps [clinically relevant] subset differences” (Parker, 2014, p. 404).

^5^A phenomenologically grounded specification of the types of some psychopathological condition is not a symptom-oriented classification, as are the classifications offered in most psychodiagnostic manuals. The latter classifications are often said to be “phenomenological” for the sole reason that they are based on the symptomatic phenotypicity of the relevant condition, as opposed to being based on etiological considerations. Here, this common clinical use of the term *phenomenology* can mislead.

^6^Ehrenberg’s “weariness of the self” does not characterize a type of depression. Rather, this notion points to a social fact that the author uses to explain certain experiences of inadequacy that psychiatry understands as “symptoms of depression.” Ehrenberg relates depression to self-management demands imposed on autonomous individuals who, in modern societies, are made responsible for being (or failing to be) successful. Although painful experiences of not being able to fulfill achievement expectations were ubiquitous in our sample, they were particularly conspicuous in those descriptions that informed our characterization of the third specifier.

^7^Indeed, there is an almost perfect concordance between the realms of functionality described by ICD-11 and the four dimensions characterized by the OPD-CA-2. This is surprising, since these two systems of conceptualizations emerged from separate traditions of thought.

^8^In order to accommodate the psychoanalytic notion that depression should be understood as a dysfunctional response to loss (Freud, 1917), Blatt (2004) distinguished between the distal and the proximal antecedents of a depressive reaction. Here, a situation representing a loss to the sufferer (proximal antecedent) is conceived to precipitate a depressive reaction against the background of a vulnerability given by certain representations of caregiving relationships (distal antecedent).

^9^The results of our idiographic analyses exclusively concern the descriptive *content* captured in the thematic bundles. Our interpretative procedure does not allow for drawing distinctions concerning the *quality* of the descriptions that constitute the accounts. This is why we did not include in our results this finding, which concerns the quality of the participants’ reports.
