# Supplementary material for: A phenomenologically grounded specification of varieties of adolescent depression
Source: Front Psychol. 2024 Feb 23;15:1322328. doi: 10.3389/fpsyg.2024.1322328 (PMC10922930; doi:10.3389/fpsyg.2024.1322328)
Supplement: Supplementary file 1 [file Data_Sheet_1.pdf]

Supplementary Material

A Phenomenologically Grounded Specification of Varieties of Adolescent Depression

H. Andrés Sánchez Guerrero*, Ida Wessing

Department of Child and Adolescent Psychiatry, Psychosomatics, and Psychotherapy, University Hospital Münster, Münster, Germany

*** Correspondence:**
HectorAndres.SanchezGuerrero@ukmuenster.de

A) Thematic Bundles

Participant #1

Bundle 1: Emptiness and the inability to connect with a world that has lost all appeal

When you are depressed, you are apathetic, powerless, and very limited in your ability to feel something, which is the reason why you find it pointless to get up every day.

Things that used to be fun are not appealing to you anymore and you feel like y no longer being able to find anything good; thus, you do not have the strength to deal with everyday activities.

When things lose their appeal, you feel indifferent and empty.

It is hard to motivate yourself, you often have the feeling that you urgently need a place to retreat, and you think that it does not make sense to carry out your routines.

You are not motivated to make contact with people.

Life does not make sense if you have to do things that you do not like or if the things you are doing seem to have no purpose whatsoever.

Bundle 2: Every movement requires a lot of effort: a sense of limited capabilities

When you are depressed, it seems impossible to complete everyday activities that appear so simple to others, which is something you can hardly convey.

Your body feels heavy, moving this body is very exhausting, and you only on certain days (partially) manage the routines of everyday life.

On bad days, you cannot do anything at all, thus, you stay in bed and stare at the ceiling; on better days, you only manage your daily routines with extreme effort and step by step.

This state cannot be compared to "normal" fatigue that everyone knows, since it is not just an intensification of otherwise common feelings.

It is hard to convey that you are just as limited as people who have a physical disease, whose impairment can everyone immediately see.

Bundle 3: Ruminations concerning one's inability to find a place in the world

When you are depressed, the things you have to do in order to find a place in the world seem unattainable, thus, there is a lot of worry about your future.

You tend to think a lot, but you cannot really articulate thoughts.

You think a lot about things that do not necessarily concern you personally, and you ask yourself whether this way of thinking should actually be regarded as a part of your personality.

You also think more emotionally and always in relation to what you do not want to do and what is negative, thus, you cannot see the benefits of possible actions from a rational point of view.

You feel that you have to deal on your own with depressing issues, and you begin to doubt seriously if you will ever be able to feel comfortable in society and find your own way.

Bundle 4: The feared irrelevance of your death

When you are depressed, you ask yourself what happens after you die and, particularly, what people whom you would leave behind may think and feel.

You fear that your death may be irrelevant to other people.

Participant #2

Bundle 1: Focusing on the sad and negative that has been imposed on you

When you are depressed, everything seems black, dark, and sad and you can see nothing good in life, only the negative sides you are confronted with.

You can no longer see the sunny side of situations, since you all the time think that it is raining.

You do not want to do anything; you stay in bed all the time and just want to harm yourself.

You focus on things that went wrong and do not cease to look for reasons why these things happened.

You experience your situation as the result of heavy losses.

You are so focused on your own sadness that you can no longer pay attention to others; you feel excluded from social groups that are important to you and like you are the only one who has to remain isolated.

You experience a kind of cramp and you cannot stop crying.

<u>Bundle 2: Closure to opportunities</u>

When you are depressed, you cannot think about looking ahead and cultivating hope.

You shut yourself off from activities that would be fun in other moments.

The possibility to communicate with people who are important to you and to feel accompanied disappears, thus, you feel alone.

You have the feeling that not even God is at your side or wants to free you from the burden of life.

<u>Bundle 3: Injury, defiance, and (identification with) destruction</u>

When you are depressed, your willingness to engage in activities depends on how people who are important to you deal with you.

You are seriously injured by some actions of significant others and focus on how to do something disruptive.

You are interested in the way in which others have tried to commit suicide and in the motives that led them to do so.

You feel like there is nothing that can get you out of this state, and you do not want to receive help or comfort.

You are not open to get out of your situation, do not cooperate, and do not allow others to tell you what to do.

You believe that any improvement can only be temporary and develop an attitude as if you want to remain in this painful state.

<u>Bundle 4: Private suffering (that seems to depend on others)</u>

When you are depressed, you do not expect that another person could ever be able to understand your suffering, since no one can personally experience the things you feel.

You cannot express your feelings in words.

You feel like too much has happened to ever be communicated.

You are ashamed to be that sad.

Depending on what others do, you get better at some point, thus, you feel like your affective state is not in your hands.

Participant #3

Bundle 1: Transformations in one's attitude towards the world and one's way of thinking

When you are depressed, you suddenly feel completely different about things that you may have liked before and you draw conclusions that reveal a change in your mindset.

It is particularly difficult to explain certain experiences and ways of thinking that may seem incomprehensible (e.g., that you feel a lot of self-hatred, feel like having to hurt or punish yourself, and want to see your own blood in order to feel yourself again).

Life seems pointless, which is quite different in non-depressive phases in which you even celebrate being alive.

Your preoccupation with death can be experienced as a conflict, even if you do not think about death in a fundamentally different way in these phases.

Bundle 2: A chaos of feelings, thoughts, and behavior and the noisy world

When you are depressed, your feelings become chaotic and alternate constantly; you feel completely bad, do not know what you are feeling, or have mixed feelings.

The world seems quite noisy, which has to do with the fact that it is quite loud within your head.

You are more sensitive, take more things to heart, and think about things you would not think about otherwise.

Depending on the intensity of the depressive phase you are in, you have the sense that you are no longer in control of yourself, not only in terms of feelings but also of behavior and communication.

In particularly bad phases, you suffer a kind of nervous breakdown and sometimes do things you have to regret afterwards; as a result, you feel like having been kicked off.

It is difficult to communicate with people who have never experienced these phases, since everything seems to be mixed up and you usually do not understand yourself and do not know what to explain.

Bundle 3: Self-imprisonment, distance from the world, and the numb body

When you are depressed, you are trapped in your thoughts and feel like you cannot be completely in the world.

Everything feels as if it has been blended out; since your head is quite full of thoughts, you can no longer perceive things properly.

Your capacity to think is limited; since your mind is full of thoughts about yourself, you cannot think about other things.

You feel as if you have died inside and as if you have completely gone away and could no longer get any feeling in your body.

Bundle 4: Inertia, need for rest, and the paradoxical reinforcement of isolation

When you are depressed, you limit yourself in your possibilities, since you predominantly need rest and want to be alone in order to try to sort out your own thoughts.

You experience your body as limp, lazy, and very tired.

It is hard to get things done, since you feel exhausted, sleep a lot, do almost nothing, and are not able to counteract this inertia.

Time goes by either much slower or much faster, as if it were hanging back or twisting while you are ruminating.

You feel misunderstood and alone, but you want to have little contact with others, since you need more quietness.

Bundle 5: Persistent stress and long-lasting recurring sadness

When you are depressed, you permanently feel stressed and recurrently experience long-lasting periods of sorrow.

You permanently feel alone and sad and have alienating suicidal thoughts.

Participant #4

Bundle 1: (Self-)exclusion from the world

When you are depressed, you are unable to get out of a situation that makes it impossible to regard yourself as a part of the world.

You feel separated from the world, misunderstood in a fundamental way, and only able to meet people who are in a similar situation.

The group context can represent a confrontation with a cheerfulness that is no longer attainable, which leads to an intensification of your social withdrawal tendencies.

You are in a different world, which has primarily to do with your own radically changed attitude.

Bundle 2: Captive in a chaotic underworld

When you are depressed, you feel like wandering around a chaotic underclass of the world and like not being able to get out of this level.

You feel like having to carry on doing things in order to get out of this situation, although you cannot see any orientation, start, or goal.

You walk long and confusing paths.

A feeling of a quiet chaos persisting in the background accompanies the sad character of the world.

Bundle 3: Loneliness and the evasion of negative sides of existence

When you are depressed, due to a hard-to-overcome restricted openness to other people, you find it difficult to get a hold on others.

It is particularly difficult to explain to other people what and how you feel, which has to do with people's not wanting to come in touch with negative aspects of life of which they are actually aware.

You find yourself in a very negative situation characterized by loneliness, helplessness, and perplexity—a situation that persists and makes it difficult to accept help.

Bundle 4: Changed (self-)positioning in relation to others

When you are depressed, you are more sensitive to the way other people perceive you.

You experience yourself as subordinate to persons who are not mentally ill and able to position themselves more confidently in the world and stand up better.

The fear that other people could condemn your frequent dysphoric mood as a sort of attention-seeking behavior reinforces your social withdrawal tendencies.

You feel that some people are taking advantage of your having collapsed into yourself.

You quickly feel judged, particularly when you are in contact with peers.

Bundle 5: A transformed relation to reality and a transformed space of possibilities

When you are depressed, you relate differently to the world, which, correspondingly, appears to you as transformed.

You fear that, if you adopt a more realistic and appreciative attitude, this may come back to you like a boomerang that worsens your situation.

Your general attitude is negative in feeling, perceiving, and thinking, which does not imply that you cannot register how reality actually is.

Bundle 6: Effort and a burden that is bodily experienced

When you are depressed, an emptiness experienced in the body does no longer allow you to achieve things you could actually achieve.

Your bodily posture and a series of bodily symptoms reveal the terrible condition in which you find yourself.

You experience yourself as forgetful and, for physical reasons, no longer able to perform activities you actually find easy.

No longer being able to do things that you could and would normally want to do is exhausting.

You feel like all your efforts lead to a sense of merely being there somehow.

Bundle 7: Being unable to get a hold on things and the peculiar desolation of depression

When you are depressed, it is hard to get a hold on things; due to your feeling that you can merely engage in unfocused and inefficient activity, you lose all motivation.

You cannot really engage with anything, which makes you feel empty.

Positive moments appear as crushed between a series of prevailing long-lasting negative sequences.

Participant #5

Bundle 1: Thin-skinnedness (extreme sensitivity to the mood and opinion of others) and the feeling of being misunderstood and alone

When you are depressed, you feel sad in a special way and like a different person; you feel particularly emotional and emphatic.

You pay more attention to other people and focus less on other things.

You can better put yourself into the shoes of persons who also look sad and seem to be in need of confirmation.

You feel that other people, particularly strangers, are stressed and in a bad mood (the way you are), which stresses you additionally, while friends look happier, which makes you angry because of the distance to your own mood.

Adults seem to get annoying faster.

You are more sensitive and disposed to feel ignored or invalidated.

You feel ridiculed by everyone and like nobody could really understand you.

You feel lonely and suffer from listlessness and fluctuating feelings.

You can only partially convey your feelings, since you do not know what it is that you are feeling and think that others cannot understand your feelings if they are not acquainted with such a condition.

Bundle 2: Powerlessness and listlessness

When you are depressed, you feel lonely and powerless.

It is hard to get involved in anything, since you have trouble concentrating, do not feel like doing things that you otherwise would like to do, and do not have the energy to do anything.

Since you feel too powerless for anything, you do nothing, even if you have set yourself up to complete a particular task.

Bundle 3: Relatively abstract rumination

When you are depressed, you are concerned with relatively abstract considerations; you think more about life and the world, generally, than in non-depressive moments, when you are more concerned with concrete everyday issues.

You can only focus on your own thoughts.

Participant #6

Bundle 1: A world that is increasingly less apt to lift you up: estrangement from an existence you cannot hold on

When you are depressed, it is always different, always worse, always longer; you progressively lose the desire to live, are less and less in this world, and just do not feel that you can be lifted up.

You feel increasingly cut off from the world and are completely on your own and no longer able to communicate with others.

If you partially manage to get out of a depressive episode, something always remains altered.

Against the background of a progressive feeling of brokenness, at some point, it does no longer make sense to project yourself into a concrete possible future.

Bundle 2: A self-loathing that becomes paranoid: self-depreciation and distrust in relations

When you are depressed, you somehow permanently have the impression that you are a burden to other people and remain imperfect.

With persons who are important to you, you get the feeling that they do not need you or that you are burdening them.

You feel useless, disgusting, and dirty and are ashamed of your own body, which you find so horrible that you cannot even look at yourself anymore.

You fight yourself down until your happiness façade falls.

You pick out situations to fuel your belief that significant others might not have a reason to stand by you, that they are probably only doing so out of pity or a sense of obligation.

You imagine that other people are talking about you, that they do not need you, and that you do not do them any good.

You have the feeling that people close to you are becoming devious and that you can no longer trust them.

Bundle 3: Emotional numbness as a defense against emotional lability

When you are depressed, you fluctuate between outbursts of anger, phases in which you feel irritable or sad for no reason, and states in which you do not feel anything at all and shut yourself off from communication as if you are emotionally numb.

In these phases of emotional numbness, you no longer experience certain ambivalences that you normally face when you suffer extreme mood swings.

You cannot feel joy, not even in situations where you want to be happy.

Bundle 4: Not willing and not being able to continue: limitations in cognitive and practical areas of life resulting from an impaired ability to will

When you are depressed, you do many things worse just because you do not want to do anything anymore and want to be (left) alone.

You are absent in the sense that you find it difficult to concentrate on life and prefer to go somewhere else in thoughts.

You do not have the strength to stand up, but this does not really come from the body, it comes from the impression that you have no reason to go through everyday life.

It becomes increasingly difficult to concentrate and your performance at school deteriorates even in subjects you like.

You experience your concentration difficulties as a kind of blockade that throws you back into your own world.

You do not want to live anymore.

Bundle 5: Unable to maintain the mask: unsustainably pretended good mood

When you are depressed, you try to hide the fact from other people's eyes that you are feeling so bad.

At some point, you feel like you can no longer use a smile as a shield to prevent others from realizing how badly you feel.

Bundle 6: Staying depressed (no matter what you do or how you feel): a particular hopelessness

When you are depressed, even in less depressive phases, you feel like depression is there and can manifest at any time.

You get the impression that it does not make any difference whether you are fighting your depression or not.

Participant #7

Bundle 1: An affective change associated with one's separation from the world

When you are depressed, you feel as if you are partly out of the world; you do not register some things and experience emotions differently.

You feel as if you have internalized a mixture of negative feelings that you can no longer differentiate.

You feel sad in a special way, which has nothing to do with the intensity of the experiences but with the strange feeling of not being in the world and only being able to think of one thing.

The world seems to be cut off from you, as if it were outside the bubble in which you are trapped.

Bundle 2: Trapped in thought loops

When you are depressed, you find yourself in a prolonged or indefinite phase in which you are trapped in increasingly sad loops of thoughts from which you can only get out with support, as these thoughts are very dominant in your mind, cannot be blended out, and keep you from sleeping and eating.

It is very exhausting to waste so many thoughts on one thing and find yourself unable to think about other things, thus, a cycle of sadness pulls you down more and more.

Although your focusing on one thing brings possible alternative thoughts to get lost, your ability to think does not seem transformed.

The world looks different in that you can feel little fun and cannot concentrate on worldly objects when you are that focused on one thing.

Bundle 3: Social distancing and the feeling of not being understood

When you are depressed, you feel particularly in social situations the effects of your prolonged sadness.

You feel the furthest away from your peers and the least understood by them; you have the feeling that you are unable to connect with them.

The best way to convey to a person how you feel is by bringing her to understand what it is that is important to you and you have lost.

Bundle 4: Inertia

When you are depressed, the body feels heavy in the sense of sluggish.

Because of a constant feeling of sadness, you do everything much slower and with less energy; you do things that you have never really found funny less enthusiastically (or do not do them at all).

Bundle 5: A changed space of (existential) possibilities: the gift of death

When you are depressed, your life does not seem important to you.

You suddenly think about your own death in terms of a possible and desired liberation (from the burden of life).

This desire for redemption or liberation from life seems particularly difficult to convey, since those who are not acquainted with phases in which you cannot help feeling oppressed usually see life [and not death] as a gift.

Participant #8

<u>Bundle 1: Emotional disability and affective confusion</u>

When you are depressed, you feel like you are emotionally impaired.

You do not know how to feel and are emotionally unstable and quickly burdened without a clear reason.

You are in a state that is long lasting and diffuse—a state you cannot explain or classify.

You do not feel a certain emotion but are in a composite affective state consisting of hopelessness, a perceived impossibility to be surprised, and the feeling of not wanting to experience the immediate future.

The affective emptiness you experience leads to a state of confusion and to the impossibility to communicate how you are doing.

<u>Bundle 2: An immutable mood and a constant impossibility to feel differently</u>

When you are depressed, there is a constant terrible feeling and you do not see the prospect of recovery at all.

You are in a mood that does not change, which you experience as an impossibility to feel different emotions.

You feel like you have lost something but do not understand what it is, thus, you do not have any hope that a grieving process could resolve this feeling.

You regard the immediate future as something that you would prefer not to experience, since you assume the persistence of your bad mood and your inability to respond to certain things.

You lose the feeling of naturalness that, in everyday life, accompanies a typical day—a day that you can simply spend doing something.

To get out of this condition, you socially withdraw, even though you cannot be alone.

<u>Bundle 3: Separation from the reality of the perceiving body</u>

When you are depressed, you feel unreal, like you are not in your body.

It is as if your thoughts were separated from your body, which is associated with the feeling of not really being there.

You feel like you are in a movie, as if you are not actually there, even though you can see what is happening in front of you.

You feel like you have some kind of filter on your eyes, as if you are looking through something.

Existence feels like a passing away of days in a row, and not like a real being aware of something.

<u>Bundle 4: Alienation from peers</u>

When you are depressed, your peers appear to you like bad people, as if they are enemies, the opposite of you.

You experience peers as people who are completely different and think wrong about you; your bodily existence is then associated with the unpleasant feeling of being present to others, while you do not want to be seen.

<u>Bundle 5: Depression as a consequence of a congestion of feelings</u>

When you are depressed, despite the difficulties to understand your feelings, you can identify factors that could have led to this state, particularly events that you have tried to overlook and can no longer change today.

You feel that repressed feelings associated with loss, loneliness, and lovesickness have accumulated and can no longer be contained.

Participant #9

<u>Bundle 1: Internal fetters and depressive pseudo-paralysis</u>

When you are depressed, you feel like trapped inside: you feel unable to move and unable to help yourself.

You feel very tired, do not want to do anything anymore, do not feel like doing anything, and, even among close people, feel alone.

You feel like you are not able to move and have no control over your own body, which you can only limitedly perceive.

You are in a state that in certain respects resembles death, but you painfully feel your body and know that you could, in principle, move it.

<u>Bundle 2: Opacity of the sources of depression, threatening uncertainty, and altered temporality</u>

When you are depressed, you feel like you have been washed away by a dark and impenetrable wave that surrounds you, drowns you, and wears you down.

It is hard to understand why you feel this way and very small events can trigger a reaction that is accompanied by a kind of panic and paralysis at the same time.

The future seems more threatening, since you do not know what is coming, tend to expect negative things, and have no goal in mind.

You get the feeling that time does not go by, but then it is somewhat gone.

You have the feeling that you are not developing at the same pace as others do.

You are in a suspended state that exhibits the character, but not the clear temporal limits, of a phase.

Bundle 3: Transformed ability to perceive

When you are depressed, your capacity to perceive completely changes and, derivatively, your capacity to think; you feel like looking through a magnifying glass when you are actually perceiving the world through a keyhole.

You tend to pay attention to what is not fulfilled, take a more extreme view of small things that go wrong, and find no longer important things that actually mean something to you.

Bundle 4: A fundamentally modified affective field

When you are depressed, you are in a state that cannot be compared to emotions or states that someone who has not experienced depression might know.

You feel a particularly intense and long-lasting discomfort that resembles sadness but is accompanied by the desire not to have to live or to go on anymore.

The part of your affective life that usually entertains compensating positive emotions is lost.

You sometimes feel an overwhelming incapacity to participate, which buffers feelings and is more bearable than the primary negative mood that prevails in worse moments when you experience yourself as more emotional.

You find yourself in a state that is difficult to convey, since the overall picture of this state cannot be reconstructed from those individual aspects of the experience that are also known to non-depressive people.

Bundle 5: Distortion of meaning, alienation from the world, and the burden of life

When you are depressed, the world seems more surreal, as if it is fake and not important.

You perceive the world as if a bad filter has been put on it.

Many things seem sharper and blurred at the same time, as if the events are less important but fundamentally more troublesome than usual.

You experience life as unbearable, more negative, and too long and not necessarily worth living; as something you have nothing to do with.

If already in life you cannot cope with yourself, the idea of death as eternity becomes a horror idea.

Bundle 6: Self-devaluation and an ambivalent relation to the social environment

When you are depressed, you experience yourself as replaceable and you have mixed feelings regarding contact with other people: you want to do more with them and, at the same time, you want to break off contact.

It is difficult to perceive and appreciate the closeness of people: you want to be left alone and isolate yourself, even though you fear the loss of important people.

You perceive people your age as more negative and less important to you, which reinforces the impulse to avoid them and to isolate yourself.

You are more irritable and less able to deal with yourself and cannot understand why someone could want to have something to do with you.

You experience adults, particularly caregivers and authority persons, as hostile or unfair, as though they are treating you from a higher position.

Bundle 7: Lack of desire and practical inability

When you are depressed, you feel a generalized lack of motivation and desire.

Due to your lack of motivation, desire, and strength, everything seems more difficult than it usually would and you can hardly perform routines.

Participant #10

Bundle 1: A permanent change in the basic tone of life and a shift in one's world-relatedness that concerns both thinking and perceiving

When you are depressed, the basic tone of life changes in a permanent way; you repeatedly return to the depressive mood whenever foreground life qualities do not override it.

Your capacity to think seems very cumbersome and sometimes no longer goal-oriented; you are preoccupied by things that would not touch you in other moments.

In the course of time, you accept a persistently twisted and limited way of perceiving as your own way of being, thus, you can only recognize the shifts in your world-relatedness through external feedback.

Bundle 2: Lost and separated from (the vitality and the light of) the environment

When you are depressed, you find yourself in a state in which you feel drawn down and lost as well as isolated from the environment, which then appears darker.

Out of your fear of negative interpersonal experiences or of your complete (social) disinterest, you withdraw.

You find yourself in a state comparable to a black hole, in which you experience yourself as lonely, desperate, lost and little present, and basically hardly able to share in the vitality and diversity of life.

You experience yourself as paradoxically alive but not vital (lost, torn, and as if disconnected).

Bundle 3: Feelings converge in an emptiness that could be better tolerated than one's negative mood but which leads to a sense of being lost

When you are depressed, coping with various negative feelings and the lack of positive qualities in affective life leads to a sense of emptiness that is experienced as a strange loss.

Negative feelings are permanently present in your experiences, but they organize themselves in a lump-like form, which makes it impossible to feel something properly.

Your numbness constitutes an ultimate attempt to dampen persistent negative feelings that cannot be suppressed (anymore).

The state of emptiness that results from your numbness seems more bearable than the negative feelings initially associated with the depressive condition.

Bundle 4: The load of a non-vital body that fails to come to the point

When you are depressed, you desperately suffer from the weight of a non-vital body that is unable to carry itself or rise itself from the ground.

The day starts with such sense of tiredness that you feel like you have to carry around heavy weights all the time in order to get the day going.

Your body feels heavy, sluggish, and foggy, as if you have ingested something noxious of which the body cannot empty itself.

You feel like you need more time to do everything, which is associated with the feeling that time has become more elastic and that nothing really comes to the point.

Against the background of your feeling, to the effect that many things that used to be unproblematic have become practically impossible, your thoughts become self-critical, depressing, discouraging, and ultimately self-destructive.

Bundle 5: A world that is not worth living in/an existence that is not worth being lived

When you are depressed, due to the loss of positive feelings, you find yourself in a cold, foggy, and gloomy world.

You feel totally stunted, depressed, and limited in the ability to feel joy and to laugh heartily, thus your existence loses those qualities that made it livable.

Bundle 6: The experience of a double divergence

When you are depressed, due to a transformed capacity to perceive and think—as if you had put on special glasses—you cannot have experiences you have previously had and you cannot know what is the case.

You try to find comfort in the idea that life has other sides, which is discrepant to the experiences you have in this state, though.

Bundle 7: Living under (imaginary) traitors

When you are depressed, you quickly feel betrayed and hurt by your impression that other persons do not want to do something with you, which often has little to do with reality but with your own limitedly reactive attitude.

Other people are often experienced as enemies, while you experience yourself as hostile towards them, which makes contact difficult, particularly with peers.

Your feeling hurt by the reactions of another person is proportional to the degree of attachment to this person.

Bundle 8: Death as a real possibility

When you are depressed, you begin to perceive death as a real and personal possibility, as opposed to a mere theoretically possible reality.

Despite the, in principle, registered openness concerning what is going to happen next, death appears to you better than the possibilities you have in this life.

Bundle 9: A state that cannot be plausibly communicated

When you are depressed, you feel like no one can understand or even believe what you are going through, since the experiences are very peculiar.

You find yourself in a state that you can only punctually compare to the states in which other persons may be—in a state that is in a certain sense unique.

With time and through reflexive examination, you can learn to put your own state of mind into words.

You can with difficulty employ usual terms to describe your condition, since they imply either too much or too little.

B) Fragment Exemplifying an Incidental Finding

To illustrate the mentioned incidental finding pertaining to the quality of the participants' reports, here is a fragment that captures an attempt to bring participant #6 to describe her experiences. Specifically, this fragment exemplifies the relatively limited capacity of both participants whose accounts were classified under the Upset (Refusing) Mode specifier to articulate in words their own experiential life.

Interviewer: […] I would like to hear what you think what depression is.

Participant: I don't know …, I've never thought about that.

I: And if you try to think about it right now?

P: I don't know. What am I supposed to think? I don't know what this is, what it is supposed to be.

I: What would you say, what distinguishes this state from other states in which people who are not depressed can be?

P: Heh?

I: What is different when you are depressed?

P: Oh, that is what you mean. I don't know … you … I don't know, that is … I don't know, that is … you want to … I don't know, that is … I don't know how to explain it.
